# Gastrointestinal parasites of red and roe deer investigated via metagenomics and histology

**DOI:** 10.1186/s13071-026-07347-8

**Published:** 2026-07-07

**Authors:** Nele Lechleiter, Judith Wedemeyer, Jessica Junker, Julia Sehl-Ewert, Timo Homeier-Bachmann

**Affiliations:** 1 Institute of Epidemiology, Friedrich-Loeffler-Institut, Südufer 10, 17493 Greifswald, Island of Riems Germany; 2 Department of Experimental Animal Facilities and Biorisk Management, Friedrich-Loeffler-Institut, Südufer 10, 17493 Greifswald, Island of Riems Germany

**Keywords:** Metagenomics, Histology, Red deer, Roe deer, Parasites, Seasonality

## Abstract

**Background:**

Some of the most common pathogens in wildlife are parasites. Since wild cervids are phylogenetically close to a lot of our livestock species, disease dynamics can arise, for example, through shared parasites. Insight into regional patterns, shaped by ecosystems and cross-species relationships, is only slowly emerging and the species-specific knowledge about lifecycle and ecology of parasites is often based on cross-sectional studies and therefore limited. Possibilities for broad and easy investigation of parasites could be the key to widen our understanding of these systems and processes.

**Methods:**

Here, shotgun metagenomics were investigated as a method for parasite detection in fecal samples of wild ungulates. The results were further validated by histopathological examination of gastrointestinal tissues.

**Results:**

The results from the two methods are in line with similar studies, and while not being identical, complement each other.

**Conclusions:**

This investigation revealed parasite composition and seasonal dynamics in two species of wild cervid red deer (*Cervus elaphus*) and roe deer (*Capreolus capreolus*).

**Graphical Abstract:**

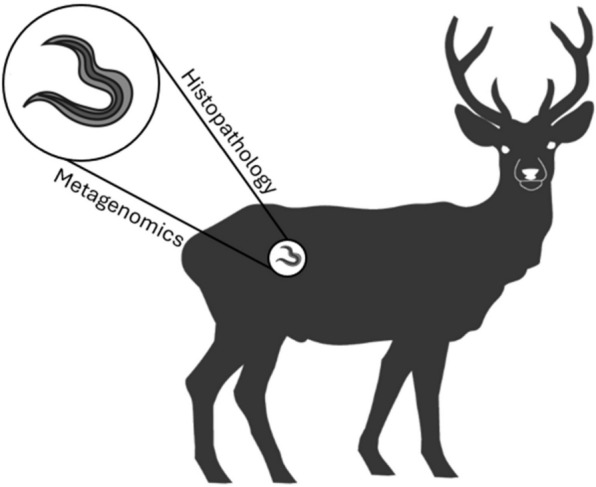

**Supplementary Information:**

The online version contains supplementary material available at 10.1186/s13071-026-07347-8.

## Background

Wild cervids are an important part of European ecosystems, a valuable game species [1], and their close phylogenetic and ecological relationship with domestic ruminants makes them potential reservoirs or bridge hosts for parasites such as *Haemonchus contortus* [2, 3]. Roe deer (*Capreolus capreolus*) specifically are the most abundant wild ungulates of central Europe [4], while red deer (*Cervus elaphus)* are some of the largest wild animals [1]. Both of these species show a population increase in recent decades and subsequently an increasing distribution across Europe [4, 5].

Some of the most common pathogens in wildlife are parasites [6]. In roe deer, for example, gastrointestinal parasites are reported as a common finding in animals sent in for necropsies, either as cause of death or secondary finding [6]. Wild and domestic animals can form multihost multiparasite systems [7], and parasitic diseases, such as some species of *Ostertagia* [8], can, through morbidity and subsequent production losses, cause great damages to livestock [9]. Therefore, knowledge about the occurrence of parasites and their composition in a wildlife host is not only of importance for conservation, but also public health. Many environmental factors, such as season or food availability [2], and individual features, such as host age, state of health, or sex [10], can have an influence on the susceptibility to parasitic disease. Consequently, it is insightful to investigate a greater number of animals to reveal infection patterns. In this study, 106 wild ungulates (28 roe deer and 78 red deer) were investigated for gastrointestinal parasites using shotgun metagenomics and histopathology. Fecal samples were chosen for a molecular investigation, as they can not only be collected with little-to-no disturbance to the animal, but also represent the shedding of parasite genetic material and potential infective stages into the environment. By comparison with the histopathological evaluation, this study further investigates the utility of metagenomic analysis for parasite detection. Parasite compositions were characterized, and influencing factors, such as age and sex of the animal and sampling season, analyzed.

## Methods

### Sampling

Samples originated from two different sampling sites. For red deer, samples were taken during routine processing within 3 h of hunting by the staff of the Deutsche Wildtierstiftung on Gut Klepelshagen in southern Mecklenburg-Western Pomerania from animals hunted in their district. Tissue samples of the abomasum, two samples of the small intestine, cecum, and colon of 3–4 cm in length were preserved in 45 ml of 4% formaldehyde in 50 ml falcon tubes (SARSTEDT AG & Co. KG, Nümbrecht, Germany) and stored at 4 °C within an hour of sampling. Fecal samples from the rectum were collected in an empty 50 ml falcon tube (SARSTEDT AG & Co. KG, Nümbrecht, Germany) and frozen at −20 °C within an hour of sampling. An additional form recording the animal ID was provided with the prelabeled sampling tubes. This ID was connected to information on the hunting date, sex, age, and weight of the animal. Samples were collected throughout 1 year, between 4 August 2023 and 8 July 2024 (Appendix 1). In this organization, the hunting season is more restricted than under state regulations, lasting until 31 December and commencing on 1 May for yearlings, 1 August for adults, and 1 September for calves. Roe deer were sampled in two regions south of Greifswald, Hegering Greifswald-Süd and the University Forest from the University of Greifswald. Hunters reached out when an animal was hunted and kept the intestines intact, which were subsequently sampled within 12 h. Tissue samples of the abomasum, small intestine, cecum, and colon of 3–4 cm in length were preserved in 45 ml of 4% formaldehyde in 50 ml falcon tubes (SARSTEDT AG & Co. KG, Nümbrecht, Germany) and stored at 4 °C within an hour of sampling. Fecal samples from the rectum were collected in an empty 50 ml falcon tube (SARSTEDT AG & Co. KG, Nümbrecht, Germany) and frozen at −20 °C within an hour of sampling. On a sampling sheet, the date of hunting, sex, and age were recorded. The sampling period lasted from the 16 April 2023 to 1 June 2023 (Appendix 2).

### Metagenomic sequencing and analysis

DNA extraction and metagenomic Illumina sequencing were executed according to Lechleiter et al. [11]. Briefly, DNA extraction was performed with the QIAamp Fast DNA Stool Mini Kit (QIAGEN, Hilden, Germany), using a slightly modified protocol. This included an additional extraction step with a TissueLyser (QIAGEN, Hilden, Germany), lysis temperature of 95 °C, and higher amount of proteinase K [12]. Samples were sent to SeqCenter (SeqCenter, Pittsburgh, USA) for Illumina sequencing on an Illumina NovaSeq 6000 sequencer (Illumina Inc., San Diego, USA), producing 2 × 151 bp paired-end reads. Library preparation was previously performed by SeqCenter, using the Illumina DNA Prep kit and unique dual indices. The reads were demultiplexed, quality controlled, and adapters trimmed by SeqCenter with bcl-convert (v4.2.4). Blanks were not included in this study, as this was an exploratory investigation with limited resources. For future studies, the use of blanks is strongly recommended, as contaminants may affect the results. The metagenomic analysis was performed using the CCMetagen pipeline [13] set to standard parameters, as described in Homeier-Bachmann et al. [14]. Within this pipeline, the tool KMA (version 1.3.0) [15] mapped reads to the NCBI taxonomic database as reference database, specifically the ncbi_nt_kma database, which excludes environmental, artificial, and unclassified sequences (https://researchdata.edu.au/indexed-reference-databases-kma-ccmetagen/1371207, accessed 15 July 2021). This is followed by a quality filtering step by CCMetagen (version 1.2.2), where the KMA output is controlled by sequence depth, coverage, and a scoring system. Closely related lineages are separated if there are SNP (Single Nucleotide Polymorphism) differences between the consensus alignments. The resulting taxonomically assigned, and quality-controlled read sets were used for further evaluations.

### Histology

Histological investigation was carried out following the work instructions of the Laboratory for Pathology at the Friedrich-Loeffler-Institut (FLI). The formaldehyde-fixed tissue was cut with a microtome blade into sections of about 5 mm in thickness. The section was enclosed in a cassette, dehydrated with an increasing concentration of isopropyl alcohol, followed by xylene as intermediary fluid, and lastly embedded in paraffin wax. Afterward, the embedded sections were cut on a microtome at around 3 μm in thickness and mounted on glass slides (VWR International, Radnor, USA). The slides were stained with hematoxylin and eosin following standard procedures. The slide then was covered with the mounting media Entellan® (Merck KGaA, Darmstadt, Germany) and a cover slip. Scanning of the slides was performed with the NanoZoomer S60 Digital slide scanner (Hamamatsu Photonics K.K., Shizuoka, Japan) and analyzed using the NDP.view 2 software (Hamamatsu Photonics K.K., Shizuoka, Japan). Analysis for parasitic infections was performed on all gastrointestinal samples. Specimen with pronounced metagenomic nematode detections were investigated further for histopathomorphological alterations of the infected tissue. The samples chosen for further inspection were ones with more than 3% reads of a nematode genus in metagenomic sequencing (W7, W8, W13, F4, F8, F10 (all *Haemonchus*), KW77 (*Onchocerca*) in comparison with three samples per species that showed a low abundance in parasitic reads (W3, F9, F15, KW25, KW35, KW46).

### Data evaluation

Data evaluation was executed in R Studio [16] in R [17], using the packages ggplot2 [18] for visualization and vegan [19] for diversity analysis. Statistics were performed on genus level and on the red deer dataset only, as roe deer were too skewed in age and sex. For alpha-diversity, Shannon Index [20], richness and Pielou’s evenness [21] were calculated and tested for differences with one-way analysis of variance (ANOVA). Groups were compared with a Student’s* t*-test, using Bonferroni correction. Differences were tested on the basis of age groups (0/1/2; age classes described in Results section), sex, or season (spring (March–May)/summer (June–August)/autumn (September–November)/winter (December–February)), while spring was not considered, due to only being represented by two samples. Beta-diversity was calculated with Bray–Curtis dissimilarity and tested using a permutational multivariate analysis of variance (PERMANOVA) with 1000 permutations. To normalize the data, the original dataset was filtered for eukaryotes, removing Viridiplantae and Fungi, and the resulting number of reads used for normalization of the parasitic reads by dividing the number of reads of a group by the total number of filtered reads to limit skewing between samples. CCMetagen uses placeholders such as “unknown phylum” if no higher phylogenetic level is available for a match on a more specific level. These assignments were only filtered if from phylum to genus no determination was provided. If at any point of the hierarchy an assignment was present, the placeholder was kept as part of the analysis.

## Results

### Sampling outcome

During the sampling period, 28 roe deer were sampled (Appendix 2), 4 of which were female. F7 and F14 were not included in the metagenomic analysis as no read data were available.

Sampling of red deer resulted in 76 animals (Appendix 1), 3 of which had to be excluded from the metagenomic analysis due to lack of metadata or parasitic reads. Animals had a weight range from 25.5 to 197 kg. Age was recorded in age classes, which differ between male and female animals. For the purpose of this study, they were pooled into three groups: class 0 for animals in their first year of life, 1 for animals younger, and 2 for all animals older than 2 years.

### Metagenomics

#### Red deer

Illumina sequencing of red deer samples produced an average of 69 million reads per sample. CCMetagen mapped an average of 24.910 reads per sample to reference sequences. KW60 and KW82 did not have metadata connected to the sample and were consequently not included in the analysis. CCMetagen did not return annotated reads for KW81.

**Fig. 1:**
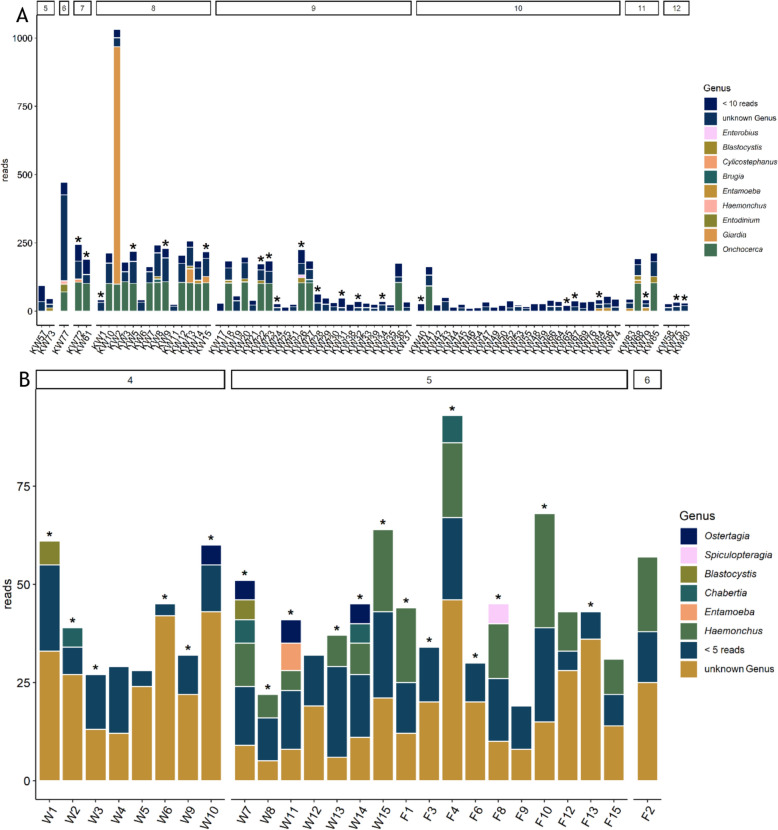
Read abundance of parasite reads in each sample, faceted by month of the year (starting with 1 (January) to 12 (December)). **A** Red deer, samples are sorted by date of hunting and faceted by season. Families with reads < 10 in a sample are summarized. Asterisks indicate that histology was positive for nematodes. **B** Roe deer, samples are sorted by date of hunting. Families with < 5 reads are summarized. Asterisk indicates that histology was positive for nematodes

On phylum level, Evosea (0.64 ± 1.12%), Nematoda (0.6 ± 1.99%), and Parabasalia (0.21 ± 0.64%) were most abundant in red deer. The genera with highest abundance were *Entamoeba* (0.30 ± 0.52%), *Blastocystis* (0.19 ± 0.37%), and *Onchocerca* (0.12 ± 0.39%) (Fig. 1A). *Giardia* showed an unusually high abundance of 1.83% in KW2, while otherwise only being present in nine other samples at less than 0.12%.

Shannon diversity and Pielou’s evenness both showed significant differences between summer and autumn, with autumn being higher in both (*P* = 0.016 and < 0.01, respectively). The evenness was significantly different between summer and winter as well (*P* = 0.027), with winter being the season with higher evenness. Regarding richness, the pattern was inverted: summer had a significantly higher richness than autumn and winter (*P* = 0.002 and 0.039, respectively). Beta-diversity according to Bray–Curtis similarity also was significantly different between autumn and summer (*F* = 7.83, *R*^2^ = 0.25, *P* < 0.001). A significant difference (*P* = 0.031) was found in richness between age groups 0 and 2, with 2 being higher.

#### Roe deer

Illumina sequencing of roe deer samples produced an average of 75 million reads per sample. CCMetagen mapped an average of 9.308 reads per sample to reference sequences. F14 did not return data from sequencing. After filtering for parasite reads, F7 was excluded since no reads were left in the dataset.

Most abundant on phylum level were Nematoda (36.77 ± 18.78%), Evosea (4.78 ± 5.6%), Parabasalia (2.5 ± 3.04%), and Apicomplexa (2.05 ± 2.81%). On genus level it was *Haemonchus* (1.97 ± 2.7%), *Chabertia* (0.55 ± 0.64%), and *Ostertagia* (0.54 ± 0.75%) (Fig. 1B).

### Histopathology

#### Red deer

Of the 78 animals that were sampled, helminths were found in 27% (21 animals). Some samples lacked certain tissues. Of the 78 animals sampled, 17 tissues out of 390 planned ones were missing. Most nematodes observed were morphologically consistent with trichostrongyloid-type worms. Some specimens showed features resembling spirurid or oxyurid nematodes; however, precise taxonomic identification would require more detailed microscopic or molecular examination. In 15 animals, parasites were observed in the abomasum; 3 in the small intestine, 2 in the cecum, and 2 in the colon (Appendix 3).

The number of sampled animals varies greatly by month of sampling (Appendix 1). In May, June, July, November, and December, less than 5 animals were sampled per month, while 14 animals were sampled in August, 28.6% of which were positive for nematodes; 24 in September, with 33.3% positive; and 26 in October, again 33.3% positive.

#### Roe deer

A total of 28 animals were sampled, 75% (21 animals) of which were positive for helminths (Appendix 3).

**Fig. 2:**
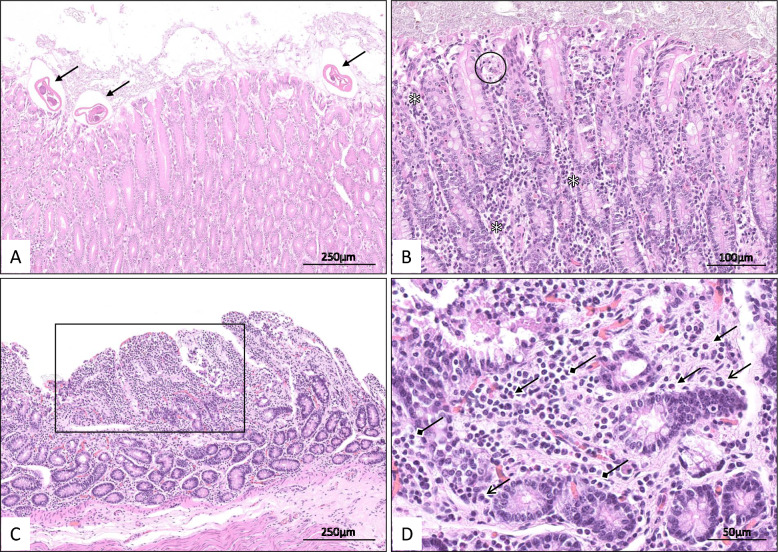
Representative histological findings of parasitic infections and associated inflammation. **A** Abomasum showing a trichostrongylid nematode cross-section (arrows), sample ID: W7. **B** Cecum with lymphoplasmacytic and eosinophilic mucosal infiltration and epithelial erosion (circle), sample ID: F15. **C** Jejunum with villous blunting and fusion (box), sample ID: F8. **D** Higher magnification of C, illustrating immune cell infiltration of the mucosa including eosinophilic granulocytes (closed arrows), plasma cells (open arrows), and lymphocytes (square arrows), sample ID: F8. All sections are stained with hematoxylin–eosin (H&E)

Most nematodes found in the abomasum were morphologically consistent with Trichostrongyloidea (e.g., *Haemonchus*, *Ostertagia* spp.). Precise identification, however, requires more detailed microscopic or molecular analysis. In total, 19 samples were positive for nematodes in the abomasum; 1 in the small intestine and 3 in the cecum. Histopathologically, animals displayed chronic active responses to parasitic infestation, such as lymphoplasmacytic and eosinophilic mucosal infiltration, in almost all samples (Fig. 2). Results of the detailed examination can be accessed in Appendix 4.

### Comparison of methodology

Histopathological analysis revealed helminth infections irrespective of the relative abundance of parasite reads detected by metagenomic sequencing. Some animals lacking metagenomic evidence of helminths showed clear histopathological signs of infection, whereas others with high read numbers were negative in histopathology. This apparent discrepancy between the two approaches was further examined in selected cases with notably high or low parasite read abundance. The complete results are provided in Appendix 4. No consistent relationship between metagenomic findings and histopathological evidence was observed.

## Discussion

Fecal shotgun metagenomic analysis detected only low relative abundances of common gastrointestinal parasites typically found in wild and domestic ungulates.

### Red deer

Finding Nematoda as one of the most abundant phyla was to be expected, as nematodes are a common finding in wild ungulates [22–24]. The histological results underlined this finding, as 27% of the animals were positive for nematodes. Since one section only displayed a very small part of the intestine, this is an indicator for a high prevalence of nematodes in the sampled animals. Trichostrongylids were the most common finding, often in the abomasum, which is in agreement with the findings of other studies on red deer in Europe [8, 23, 25]. An abomasal parasite of red deer is *Haemonchus* sp. [22], which was present in smaller abundances in the metagenomics dataset. More unusual is the most abundant nematode genus in metagenomics being *Onchocerca*. Although found in red deer in Europe, these vector-borne Filarids typically live in the subcutaneous tissues of their hosts [26]. Possibly, DNA of the organism reached the gastrointestinal tract through blood or by ingestion when the host was grooming. Another explanation could be a false assignment, the details of which will be discussed in the limitations. An interesting factor that supports the relevance of this finding is the seasonality visible in the data. Reads of *Onchocerca* are most prevalent in summer and early autumn. This pattern agrees with a disease transmitted by insect vectors [26]. Regarding alpha- and beta-diversity, summer (June–August) and autumn (September–November) differed significantly in parasite assemblage. Summer showed a greater overall richness, indicating that the number of genera peaks in summer, possibly in accordance with diversity of possible infection routes. However, when considering abundance, diversity is highest in autumn, as shown by higher Shannon diversity and Pielou’s evenness. In young red deer, strongyle egg output was found to be lower in autumn [27]. This finding was hypothesized to result from reduced propagule output rather than from parasite burden. An investigation on red deer on the Isle of Rum in Scotland revealed that the seasonality of helminth infections is species dependent, with some species showing higher abundances in summer and autumn but significant individual influences [28]. The observed diversity in autumn can therefore be the result of a specific taxon composition that is most prevalent in that time but may also be connected to the rutting season, which takes place in September–October. This time is very energy demanding, especially for males, and may suppress the immune system [29, 30], predisposing the animals to an infection. *Entamoeba* is a parasite protist genus of species with varying pathogenicity [31], which has been found in many vertebrate species, including roe deer [32]. Similarly, *Blastocystis* is a common finding in domestic animals [33] and has been shown in red deer on the Iberian Peninsula [34]. One animal showed a high abundance of *Giardia*, while not presenting any other distinctive results. This genus is present at a prevalence of 1.7–18% in red deer in Poland [35], thus finding 1 animal in 78 with an increased read count, pointing to a possible infection, is plausible. As these protist genera can cause zoonotic disease but often exist asymptomatically [36], it is of interest of public health to identify occurrences and distribution in wildlife.

### Roe deer

As in red deer, Nematoda were present as one of the most abundant phyla, which is in agreement with literature [2, 22], where one study even finds nematode prevalence up to 100% in roe deer [37]. On genus level, *Haemonchus*, *Ostertagia*, and *Chabertia* stood out, all of which have been observed in roe deer [8, 22, 37] before. Another abundant phylum was that of Apicomplexa, a group of protists, which includes pathogenic organisms such as *Eimeria*, *Cryptosporidia*, and *Sarcocystis*, all of which have been found in roe deer before [35]. No specific genus stood out though, indicating a high diversity of Apicomplexa with overall low abundance of genera. It is worth noting that in metagenomics as well as in histology, there was a higher prevalence and abundance of nematodes than in red deer. The metagenomic analysis generally showed about ten times more normalized parasite reads in roe deer than red deer, which is due to an unexplained higher ratio of host reads in the total eukaryotic reads in the red deer dataset. However, even when accounting for this imbalance, Nematoda still stand out in roe deer, which is in accordance with more positive samples in histology (27% in red deer, 75% in roe deer). A plausible explanation for this is that roe deer were sampled from mid-April through May, which is the season of parturition for females [38] and territorial establishment for males [39]. Additionally, 18 of the animals were yearlings, who have been found to have a higher parasite load than adults [40]. These factors are influential on receptiveness to parasitic infections [41], which can explain the observed intensity.

As the two species were sampled at different times and localities, direct comparisons would not be reasonable. In future studies, utilizing the ease of a metagenomic approach, it would be interesting to compare them in the same habitat, as the difference in diet, social structure, and behaviour would be expected to influence the composition and abundance of parasites [8, 22, 41].

## Comparison and limitations

Using metagenomics and histology allowed for a two-sided approach to the investigation of parasite composition in wild ungulates. The sequencing approach has the advantage of noninvasive accessible sampling material, ease of sample preparation, and a wide range of information. Several studies have used metagenomic sequencing to assess not only parasites, but also microbiota or diet of the investigated animal [42, 43]. To attribute a taxon to the reads produced by sequencing, they are aligned against reference sequences in a database. This can produce false assignments if the reference sequences are contaminated [9] or the read matches part of another sequence, which can especially happen in eukaryotic organisms with large genomes [44]. Additionally, the specific organism may not yet be included in the reference database. Contaminations from processing may likewise lead to false assignments, either skewing the results or even producing false positives. Future studies should therefore include blanks to account for the possibility of contaminants. Parasites, if present, make up only one part of the inhabitants of a vertebrate gastrointestinal tract and host and dietary DNA constitute a great part of the total eukaryotic reads. Consequently, this investigation is based on a relatively small number of reads, although the sequencing depth was high. The removal of human- and host-derived DNA from the dataset before annotating should be considered in future studies to increase the comparability of read numbers. One important caveat of both methods is that the absence of an organism, either a molecular signature or a histological cut, is not indicative of an absence in the sampled animal. Parasites in the intestine will not necessarily shed DNA or reproductive units at a given point in time [27] and if they do, its appearance in the 0.4 g of fecal matter used for DNA extraction is a chance event. In comparison with a standard polymerase chain reaction (PCR) assay, histological sections had a 21% lower detection probability investigating sarcocystis in beef [45]. These factors can explain the discrepancy between samples with high parasite read numbers but no detection in histology and vice versa. Due to the limitations discussed above, particularly the low read count, conclusions as to the parasite load and prevalence in the populations cannot be made. The significance of both methods in assessing the parasite prevalence in populations is therefore limited. Instead, this investigation focuses on an analysis of the parasite composition and dynamics. Regarding costs and effort, investigations such as these would mostly pay off if more than just one topic is being investigated. In the case of metagenomics, the data can also be used for investigating pathogens and resistance genes in the microbiome [11]. The advantage of complementing molecular data with a histological approach is not only that the detection of a parasite in the tissue is direct evidence for its presence, but also the ability to draw conclusions as to the life stage the organism is at. Additionally, further investigation of histopathological signs in the tissue can reveal physiological aspects such as an immune response in a host or the absence thereof and thus allow for conclusions as to the health of the sampled animal. Despite certain limitations, the overall results of this paper, similar to the parasite composition and seasonal effects, are in accordance with each other and those of investigations using methods established in parasitology. For studies on deer, coproscopical methods such as sedimentation-flotation are common [27, 28], as well as the inspection of gastrointestinal contents, and morphological determination of detected parasites [8]. PCR [24] and metabarcoding [46] are more established molecular approaches. Where the immediate processing of samples is possible and preservative measures are therefore not needed, established parasitological techniques such as coproscopy would offer more standardized and reliable insight.

## Conclusions

Red deer samples across one year revealed seasonal dynamics in the molecular data, with autumn being the season with highest parasite diversity. Both red and roe deer showed parasite assemblages that agree with previous data on these species. Since the two species were sampled at different locations and times, this study was not intended for a comparison. Still, it is intriguing that roe deer, which were sampled at the time of parturition, showed a greater amount of positive histopathology samples and nematode reads. In the future, comparisons of sympatric species over the course of a year could bring insights into species-specific dynamics. When respecting methodical caveats, metagenomic sequencing can be an appropriate tool for such investigations, producing results comparable to other investigations. Further complementing of molecular results can be achieved by a histopathological examination, although detection probability is lower and greater sampling and preparation effort is required. It offers the advantage of providing definite evidence for parasite presence and an evaluation of life stages and pathological context. Furthermore, a single method inherently risks overlooking certain aspects of parasite detection, a limitation that can be mitigated through the use of a complementary technique. In the future, established molecular methods, such as PCR-based approaches, could be also investigated to substantiate metagenomic results.

## Supplementary Information

Additional file 1. Red deer sampling results.

Additional file 2. Roe deer sampling results.

Additional file 3. Histopathological findings, crossed out cells indicate missing samples through falsely sampled tissues.

Additional file 4. Histopathological findings in selected samples in relation to metagenomic results. Missing samples are cases of falsely sampled tissues.

## Data Availability

Sequencing data are public under the accession numbers PRJEB90179 and PRJEB81356.
